# Chemical application improves stress resilience in plants

**DOI:** 10.1007/s11103-025-01566-w

**Published:** 2025-03-19

**Authors:** Khurram Bashir, Daisuke Todaka, Kaori Sako, Minoru Ueda, Farhan Aziz, Motoaki Seki

**Affiliations:** 1 Plant Genomic Network Research Team, RIKEN Center for Sustainable Resource Sciences, 1-7-22 Suehiro-cho, Tsurumi-ku, Yokohama, Kanagawa 230-0045 Japan; 2 Department of Life Sciences, SBA School of Science and Engineering, , Lahore University of Management Sciences, DHA Phase 5, Lahore, Pakistan; 3 Department of Advanced Bioscience, Faculty of Agriculture, Kindai University, Nakamachi, Nara 3327-204 Japan; 4 Plant Epigenome Regulation Laboratory, RIKEN Cluster for Pioneering Research, 2-1 Hirosawa, Wako, Saitama Japan; 5 Kihara Institute for Biological Research, Yokohama City University, Yokohama, Japan; 6 Graduate School of Science and Engineering, Saitama University, Saitama, Saitama Japan

**Keywords:** Acetic acid, Chemical priming, Heat tolerance, Drought tolerance, Ethanol, Metabolic reprogramming

## Abstract

In recent years, abiotic stresses, including droughts, floods, high temperatures, and salinity, have become increasingly frequent and severe. These stresses significantly hinder crop yields and product quality, posing substantial challenges to sustainable agriculture and global food security. Simultaneously, the rapidly growing global population exacerbates the need to enhance crop production under worsening environmental conditions. Consequently, the development of effective strategies to strengthen the resilience of crop plants against high temperatures, water scarcity, and extreme environmental conditions is critical for mitigating the impacts of abiotic stress. Plants respond to these environmental challenges by reprogramming their transcriptome and metabolome. Common strategies for developing stress-tolerant plants include screening germplasm, generating transgenic crop plants, and employing genome editing techniques. Recently, chemical treatment has emerged as a promising approach to enhance abiotic stress tolerance in crops. This technique involves the application of exogenous chemical compounds that induce molecular and physiological changes, thereby providing a protective shield against abiotic stress. Forward and reverse genetic approaches have facilitated the identification of chemicals capable of modulating plant responses to abiotic stresses. These priming agents function as epigenetic regulators, agonists, or antagonists, playing essential roles in regulating stomatal closure to conserve water, managing cellular signaling through reactive oxygen species and metabolites to sustain plant growth, and activating gluconeogenesis to enhance cellular metabolism. This review summarizes recent advancements in the field of chemical priming and explores strategies to improve stress tolerance and crop productivity, thereby contributing to the enhancement of global food security.

## Key message

Chemical priming presents a novel approach to enhancing plant growth and stress tolerance both spatially and temporally by modulating plant metabolism through transcriptomic and epigenetic changes.

## Introduction

In recent years, environmental stresses such as droughts, high temperatures, floods, and salinity have increasingly threatened sustainable crop production and global food security. The escalating demand for food, driven by the rapidly growing global population, adds complexity to the challenge of boosting crop production under worsening environmental conditions (Hickey et al. 2019). Different crops and varieties exhibit varied responses to environmental stresses, including drought, heatwaves, and salt stress (Zhang et al. 2018; Weiszmann et al. 2023). Among these, drought stress is particularly detrimental to crop production as it impedes plant growth and development (Gupta et al. 2020; Zhang et al. 2022b).

Climate change is expected to exacerbate the frequency and severity of droughts, floods, and high temperatures, posing further threats to crop productivity (Yeung et al. 2019; Ault 2020; Tellman et al. 2021; Warren et al. 2022). Excessive heat amplifies these challenges by increasing evaporation and transpiration rates, resulting in substantial water loss and restricted plant growth (Mills et al. 2018). Simulated models predict significant declines in crop yields due to rising temperatures; for instance, maize yield is projected to decrease by up to 24% by the end of the century (Jägermeyr et al. 2021; Warren et al. 2022).

Soil salinity is another critical factor adversely affecting plant growth and development, ultimately reducing crop yields. High levels of NaCl limit water availability and disrupt cellular metabolism, as the accumulation of sodium and chloride ions proves toxic to plants (Van Zelm et al. 2020). Consequently, understanding plant responses to high temperatures, salinity, and limited water availability is essential for developing crop varieties better adapted to environmental stresses, thereby improving crop productivity under changing environmental conditions (Bailey-Serres et al. 2019; Bashir et al. 2019; Hammer et al. 2020).

Plants respond to environmental changes by reprogramming their transcriptome, which subsequently regulates the cellular metabolome (Rasheed et al. 2016; Fàbregas and Fernie 2019). These environmental shifts often induce oxidative stress, compelling plants to rely on anaerobic fermentation to produce compounds such as ethanol and acetic acid (Ismond et al. 2003; Oliver et al. 2009; Kim et al. 2017). The transcriptomic and metabolic adjustments plants make in response to environmental stresses are reasonably well understood, and various strategies have been proposed to enhance plant tolerance to these conditions (Todaka et al. 2024; Gupta et al. 2020; Kuromori et al. 2022; Baekelandt et al. 2023).

Developing improved plant genomes through classical breeding, transgenic technologies, or gene editing is often a time-intensive process. In recent years, however, chemical treatment has emerged as a promising approach for enhancing plant growth and yield, offering a viable solution to bolster food security under ever-changing environmental conditions (Savvides et al. 2016; Vaidya et al. 2019; Sako et al. 2021b; Spanos et al. 2021; Sheikhalipour et al. 2022; Wang et al. 2022; Panahirad et al. 2023; Sheikhalipour et al. 2024).

**Fig. 1:**
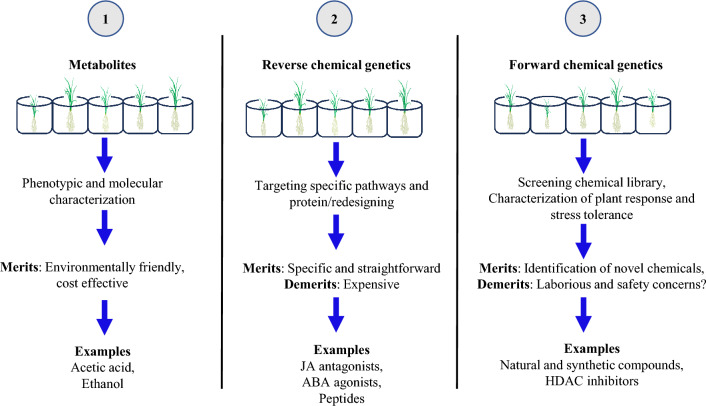
Chemical treatment strategies for enhancing abiotic stress resilience in plants

Chemical treatments provide an opportunity to temporally and spatially regulate the transcriptome and cellular metabolome, allowing for targeted responses as needed (Kim et al. 2017; Nguyen et al. 2017; Hagihara et al. 2019; Vaidya et al. 2019; Bashir et al. 2022; Lozano-Juste et al. 2023). Broadly, chemical treatment strategies can be categorized into three groups: (1) utilizing plant metabolites as chemical priming agents; (2) employing reverse chemical genetics to design agonists and antagonists for known target proteins or pathways; and (3) using forward chemical genetics to screen chemical libraries for compounds that improve stress tolerance (Fig. 1). This review summarizes recent advances in chemical treatment applications for crop improvement and discusses sustainable strategies to enhance environmental stress tolerance and crop production, thereby reinforcing food security.

## Chemical treatment strategies

Improving crop production to ensure food security is critical in addressing the challenges posed by an expanding population and deteriorating environmental conditions (Godfray et al. 2010; Hickey et al. 2019). Chemical treatment and priming have emerged as sustainable solutions for enhancing crop production under changing environmental conditions. Both forward and reverse chemical genetic approaches are employed to identify chemicals that mitigate damage under stress conditions (Serrano et al. 2015). Forward chemical genetics involves screening chemical libraries to identify candidates that improve plant growth under specific stress conditions (Fig. 1). Libraries such as the LOPAC Pfizer chemical library (Sigma-Aldrich; (Toh et al. 2018)), ITbM (Toh et al. 2018; Sako et al. 2021c), the Redox Library (Enzo Life Sciences; (Toh et al. 2018)), NPDepo (Kato et al. 2012; Sako et al. 2020), the library of active compounds on Arabidopsis (Zhao et al. 2007; Sakai et al. 2017), and the PubChem chemical libraries (Wang et al. 2017), comprising a vast collection of chemicals, have been utilized.

The ideal characteristics of these chemicals include small molecular size, membrane permeability, efficacy at lower concentrations, selective biomolecular interactions to minimize side effects (Smukste and Stockwell 2005; Serrano et al. 2015), environmental safety, ease of synthesis, and affordability for large-scale applications. The screening process is labor-intensive, involving the cultivation of a large number of plants or tissues under specific stress or environmental conditions, followed by treatment with numerous small chemicals (Toh et al. 2018; Sako et al. 2021b). Through these screens, several novel candidates have been identified that mitigate stress and enhance plant growth (Ziadi et al. 2017; Sako et al. 2020). Once target proteins are identified, these chemicals can be modified to improve their interaction with the target proteins, increasing binding efficacy while minimizing nonspecific interactions (Kinoshita et al. 2021). Virtual screenings based on in silico testing of protein/chemical interactions are not recommended for the forward chemical genetic approach, as the target proteins or pathways are typically unknown (Fig. 1).

The reverse chemical genetics approach, in contrast, targets chemicals that interact with a specific protein. In this method, virtual screening is beneficial for narrowing down potential candidates that may interact with a specific protein (Kinoshita et al. 2021). Designing agonists or antagonists to regulate stress responses effectively can significantly enhance plant resilience to various stresses (Takaoka et al. 2018; Vaidya et al. 2019; Vaidya et al. 2021; Hayashi et al. 2023). Reverse chemical genetic screens are particularly efficient for identifying chemicals that regulate specific metabolic or signaling pathways, such as ABA agonists that control stomatal opening (Vaidya et al. 2019; Li et al. 2021; Vaidya et al. 2021; Vaidya and Cutler 2022; Lozano-Juste et al. 2023; Roeder et al. 2023) or agonists for COI1-JAZ complexes that regulate immune responses (Takaoka et al. 2018; Hayashi et al. 2023). However, this approach may be less suitable for identifying chemicals that interact with proteins whose cellular functions are poorly defined.

## The effect of chemical agents to induce stress tolerance

Chemical treatment strategies have been successfully employed to enhance plant growth and stress tolerance. Compounds such as acetic acid, ethanol, aspartic acid, and 5-aminolevulinic acid induce a broad spectrum of changes, including the regulation of reactive oxygen species (ROS) production and scavenging, thereby enhancing stress tolerance (Li et al. 2011; Nguyen et al. 2017; Sako et al. 2021b, 2021a; Das et al. 2022; Helaly et al. 2022; Rahman et al. 2022; Sadak et al. 2022; Ghosh et al. 2024).

Chemical agents improve photosynthesis, promote plant growth, and enhance tolerance to drought, heat, cold, and other stresses by modulating metabolic, molecular, physiological, and morphological processes. The efficacy of these agents in mitigating specific stresses depends on the duration and method of application, dosage, and frequency of treatment. These factors are critical for determining the scalability of a particular chemical to support socioeconomically sustainable food security. This review explores how different chemicals enhance stress resilience in plants.

## Naturally existing plant metabolites

Plant metabolism, a complex network of biochemical reactions, supports plant growth, development, and responses to various environmental conditions. Genetic diversity and genome-environment interactions influence plant metabolism by driving transcriptomic changes and the synthesis of primary and secondary metabolites. Environmental factors such as light, temperature, water availability, redox status, and nutrient levels modulate metabolic pathways in response to specific conditions.

For instance, under drought stress, plants accumulate osmoprotectants, reduce photosynthetic capacity, and fine-tune primary and secondary metabolic processes (Matsui et al. 2008; Shinozaki and Yamaguchi-Shinozaki 2022; Zhang et al. 2022b). Exogenously applied plant metabolites such as acetic acid, ethanol, and nicotinic acid have been shown to regulate plant growth and enhance stress tolerance across various crop species (Kim et al. 2017; Utsumi et al. 2019; Ahmad et al. 2021; Ogawa et al. 2021; Bashir et al. 2022).

## Acetic acid, ethanol, and other metabolites

**Fig. 2:**
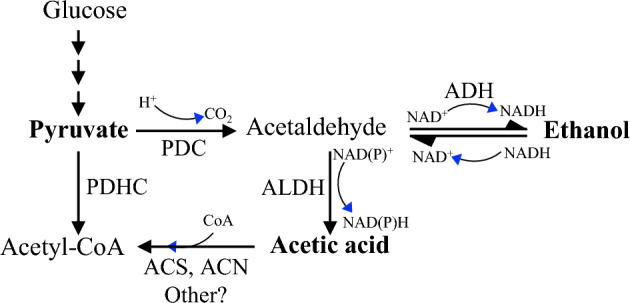
Ethanol and acetic acid biosynthesis pathways are conserved in plants and moss Pyruvate is converted to acetyl-CoA by the pyruvate dehydrogenase complex (PDHC). Under anaerobic and stress conditions, pyruvate is metabolized into acetaldehyde by pyruvate decarboxylase (PDC). Acetaldehyde can then be reversibly converted into ethanol. Alternatively, acetaldehyde may be oxidized to acetic acid, which is subsequently transformed into acetyl-CoA either in the chloroplast via acetyl-CoA synthetase (ACS) or in the peroxisome through ACN1.

Acetic acid, an organic acid, is responsible for the pungent smell and tart taste of vinegar. In plants, acetic acid is biosynthesized from pyruvate and shares a metabolic pathway with ethanol (Kim et al. 2017). Commercially, vinegar is produced through microbial fermentation of sugars and starches and is widely recognized for its health benefits (De Roos and De Vuyst 2018). Ethanol, also known as ethyl alcohol, is a colorless, volatile liquid commonly used as a fuel additive, organic solvent, and disinfectant. Biologically, ethanol is synthesized through anaerobic fermentation (Bui et al. 2019). The process begins with the decarboxylation of pyruvate to form acetaldehyde (Rasheed et al. 2018), which is then reduced to ethanol through the addition of hydrogen atoms from NADH and H⁺. Ethanol can also be oxidized back to acetaldehyde and further metabolized into acetic acid (Fig. 2).

Studies have demonstrated that the application of low concentrations of acetic acid and ethanol increases abiotic stress tolerance in plants. Ethanol treatment notably enhances salinity stress tolerance in Arabidopsis and rice (Nguyen et al. 2017). Transcriptomic analyses indicate an upregulation of ROS-related genes following ethanol treatment. This treatment also increases ascorbate peroxidase activity, facilitating the conversion of H₂O₂ into H₂O. DAB (3,3′-Diaminobenzidine) staining, a method for visualizing ROS accumulation, shows a reduction in ROS levels after ethanol treatment, highlighting its role in improving salinity stress tolerance in Arabidopsis and rice (Nguyen et al. 2017).

Ethanol treatment also enhances drought stress tolerance in Arabidopsis (Bashir et al. 2022) and other crops (Vu et al. 2022; Bashir et al. 2022). In Arabidopsis, ethanol-induced drought tolerance involves multiple adaptive mechanisms, including stomatal closure, reduced water loss, and the accumulation of metabolites such as sugars, amino acids, and glucosinolates (Bashir et al. 2022). NMR analyses have revealed that ethanol is converted into sugars through gluconeogenesis, which plays a significant role in enhancing drought stress tolerance (Bashir et al. 2022). In cassava, a critical tropical crop, ethanol treatment induces ABA accumulation and stomatal closure, reducing transpiration and improving drought tolerance (Vu et al. 2022).

In soybean, ethanol application enhances growth under salt and drought conditions by improving physiological parameters, including photosynthetic pigment content, ROS detoxification, net photosynthetic rate, shoot relative water content, water use efficiency, and the levels of K⁺ and Mg^2^⁺ (Das et al. 2022; Rahman et al. 2022). Furthermore, ethanol application has been shown to increase heat stress tolerance (Matsui et al. 2022; Todaka et al. 2024). Under high temperatures, ethanol-treated Arabidopsis plants exhibit higher survival rates compared to water-treated plants, an effect attributed to the activation of the Unfolded Protein Response (UPR) (Matsui et al. 2022). Similar results have been observed in field-grown lettuce (Matsui et al. 2022). Likewise, ethanol treatment enhances cold stress tolerance in sorghum (Ghosh et al. 2024).

Under high-light conditions, ethanol treatment increases the activity of antioxidant enzymes and upregulates genes involved in flavonoid biosynthesis in Arabidopsis (Sako et al. 2021a). These changes lead to reduced ROS accumulation and decreased photodamage.

**Fig. 3:**
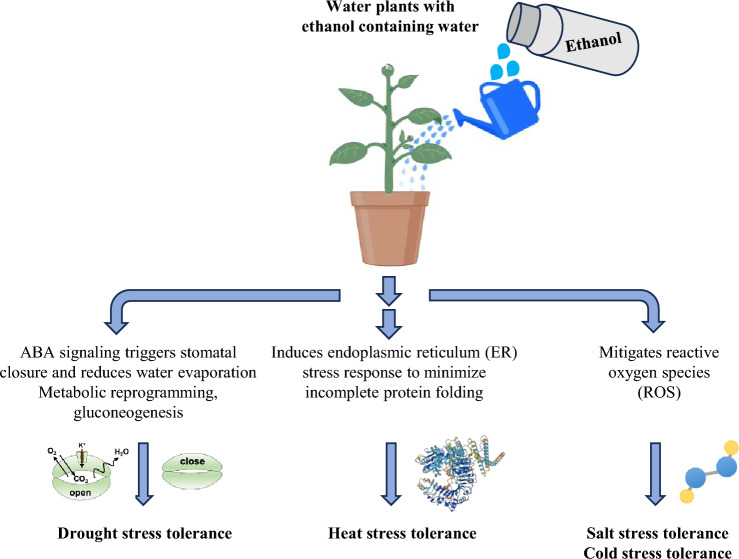
Proposed molecular mechanisms of ethanol-mediated stress tolerance in plants. The key mechanisms underlying ethanol-mediated tolerance to drought, salinity, heat, and cold stresses are summarized.

These findings underscore the effectiveness of ethanol in enhancing tolerance to various abiotic stresses across different plant species (Fig. 3). Additionally, ethanol has gained significant attention as a renewable and eco-friendly alternative to fossil fuels (Tyner 2008). Bioethanol, derived from crops such as cassava, corn, sorghum, and sugarcane (Tse et al. 2021), contributes to carbon neutrality and agricultural sustainability, thereby strengthening food security.

Treating plants with acetic acid and ethanol not only enhances drought stress tolerance but also improves heat and salt stress tolerance across various crop species (Kim et al. 2017; Nguyen et al. 2017, 2023; Utsumi et al. 2019; Ogawa et al. 2021; Sako et al. 2021b; Vu et al. 2022; Matsui et al. 2022; Bashir et al. 2022; Rahman et al. 2024). These treatments induce alterations in the plant transcriptome and metabolome in response to different metabolites (Kim et al. 2017; Matsui et al. 2022; Bashir et al. 2022), including epigenetic modifications that regulate these processes (Kim et al. 2017). External application of acetic acid, particularly through roots, promotes jasmonic acid (JA) synthesis and histone H4 acetylation, enhancing drought stress tolerance in crops such as rice, maize, rapeseed, and wheat (Kim et al. 2017; Ogawa et al. 2021). Furthermore, increased acetyl-CoA levels enhance histone acetylation, which may benefit plants under diverse environmental conditions (Chen et al. 2017).

Acetic acid and ethanol are rapidly converted into acetyl-CoA, a crucial metabolite for fatty acid biosynthesis and the tricarboxylic acid (TCA) cycle (Oliver et al. 2009). NMR analyses have demonstrated that root and shoot tissues metabolize ethanol and acetic acid efficiently, with ^13^C-labeled metabolites such as citrate, succinate, malate, and aspartic acid detected after treatment with labeled ethanol (Bashir et al. 2022). Interestingly, labeled putrescine is observed exclusively in root tissues following ethanol treatment, suggesting independent metabolism of ethanol in roots and shoots (Bashir et al. 2022). Putrescine biosynthesis plays a critical role in plant stress responses, and its suppression increases drought sensitivity, which can be reversed through exogenous application of putrescine (Wu et al. 2016).

Labeled glucose and fructose have been detected in both root and shoot tissues after treatment (Bashir et al. 2022). Additionally, labeled derivatives of glycolysis, including choline, ethanolamine, glycerate, and glycerol, were identified. Choline contributes to the synthesis of membrane lipid phosphatidylcholine (PC) and serves as a precursor for glycine betaine, an osmoprotectant essential for coping with various environmental stresses (Rontein et al. 2001; Annunziata et al. 2019). These findings suggest that, like ethanol, acetic acid is metabolized into sugars via gluconeogenesis, potentially supporting plant growth under stress conditions (Bashir et al. 2022).

Contrasting effects of acetic acid and ethanol treatments have been observed, with acetic acid more adversely affecting seed germination and plant growth than ethanol (Matsui et al. 2022; Bashir et al. 2022). In Arabidopsis, ethanol treatment reduces stomatal aperture, whereas acetic acid does not (Kim et al. 2017; Bashir et al. 2022). Additionally, ethanol treatment does not activate the JA signaling pathway, a key mechanism for drought stress tolerance (Kim et al. 2017; Bashir et al. 2022). These findings highlight distinct molecular mechanisms underlying ethanol- and acetic acid-induced drought stress tolerance.

The processes of fermentation and gluconeogenesis are critical for helping plants adapt to stress by modulating cellular metabolism. During alcoholic fermentation, pyruvate is decarboxylated to acetaldehyde, which is subsequently converted into ethanol, regenerating NAD⁺ and sustaining glycolysis and other cellular processes. This pathway is conserved across moss, fungi, and plants but is absent in animals (Kim et al. 2017; Rasheed et al. 2018). Acetic acid and ethanol are quickly converted into acetyl-CoA, which is essential for epigenetic regulation, fatty acid biosynthesis, and the TCA cycle (Lin and Oliver 2008; Oliver et al. 2009). The site of acetyl-CoA biosynthesis is critical, as this metabolite cannot cross membranes (Lin and Oliver 2008).

In peroxisomes, acetate is converted into acetyl-CoA, participating in the glyoxylate cycle, which is a more efficient carbon utilization pathway than the TCA cycle as it does not release CO₂. NADH production during the glyoxylate cycle further supports metabolic enhancement in plants. While the roles of the glyoxylate cycle and gluconeogenesis in seed germination are well-established (Eastmond et al. 2000, 2015; Cornah et al. 2004), their functions during vegetative growth and abiotic stress responses require further investigation.

Externally applied ethanol and acetic acid may contribute to the glyoxylate cycle for sugar synthesis, thereby regulating cellular metabolism (Bashir et al. 2022). Sugar accumulation through gluconeogenesis could play a pivotal role in the mechanisms underlying ethanol- and acetic acid-mediated drought stress tolerance and overall plant growth. Accumulated sugars are essential for stress tolerance, and the catabolism of carbohydrates and oxaloacetate production in peroxisomes help maintain the TCA cycle. This synergy between peroxisomes and mitochondria supports energy and amino acid production, particularly during daylight hours, to regulate stomatal aperture and cope with heat stress (Korte et al. 2023).

## Amino acids

Amino acids, comprising both amino (–NH₂) and carboxyl (–COOH) functional groups along with a distinct side chain (R group) for each type, are organic compounds essential in biological processes. Proteinogenic amino acids serve as the foundational components of proteins, while non-proteinogenic amino acids do not naturally integrate into protein structures. Over 800 natural amino acids have been documented in the literature (Xue et al. 2018). Under abiotic stress, the metabolism of certain amino acids is altered, enabling them to act as signaling molecules or intermediaries in biosynthetic pathways, thereby facilitating plant adaptation to environmental changes.

Numerous studies have demonstrated that the application of proteinogenic amino acids, including asparagine, aspartic acid, glutamic acid, lysine, cysteine, leucine, phenylalanine, proline, and tryptophan, enhances abiotic stress tolerance (Table 1) (Asgher et al. 2022; Atteya et al. 2022; Jiang et al. 2022; Kim et al. 2022; Sadak et al. 2022; Liu et al. 2023). These amino acids mitigate stress-induced damage by functioning as compatible solutes under osmotic stress, maintaining pH, ion, and redox homeostasis, and serving as nitrogen or carbon reserves (Hasanuzzaman et al. 2019). For instance, aspartic acid application improves plant tolerance to salt and heat stress (Lei et al. 2022; Sadak et al. 2022). It activates antioxidant mechanisms and promotes compatible solute accumulation, reducing reactive oxygen species (ROS) and enhancing salt stress tolerance in wheat (Sadak et al. 2022). However, the precise molecular mechanisms through which amino acids activate the ROS detoxification system remain unclear.

**Table 1:** Plant metabolites improving abiotic stress resilience in plants

Compound	Stress	Plant species	Application method	Mechanism	Concentration	Time	References
Acetic acid	Drought	Arabidopsis, begonia, cassava, maize, rapeseed, rice, soybean, wheat, common bean	Pot irrigation, leaf spray	Stimulates jasmonate signaling pathway; increases the expression of ABA signaling genes; maintains ROS homeostasis; increases photosynthetic activity	10–30 mM	3–5 days	(Kim et al. 2017; Rasheed et al. 2018; Utsumi et al. 2019; Ogawa et al. 2021; Rahman et al. 2021; Allen and Allen 2021; Nguyen et al. 2023)
	Salt	Mung bean	Leaf spray	Enhances proline, Ca^2+^, Mg^2+^ accumulation; mitigates H_2_O_2_	20 mM	1 spray, 30 mL/pot	(Rahman et al. 2019)
Ethanol	Drought	Arabidopsis, cassava, rice, wheat	Pot irrigation	Modulates sucrose and starch metabolism; regulates gluconeogenesis and ABA signaling	5–173 mM	3–8 days	(Rahman et al. 2022; Vu et al. 2022; Bashir et al. 2022)
	Heat	Arabidopsis, lettuce, tomato	Pot irrigation	Stimulates unfolded protein response; enhances expression of stress related genes and gluconeogenesis	20 mM	3 days	(Matsui et al. 2022; Todaka et al. 2024)
	High light	Arabidopsis	Liquid culture assay	Reduces H_2_O_2_ production	10 mM	2 weeks	(Sako et al. 2021a)
	Salt	Arabidopsis, rice	Pot irrigation	Reduces ROS production	0.3–0.6%	1 day	(Nguyen et al. 2017)
	Cold	Sorghum	Pot irrigation	Reduces ROS production	0.3–0.6%	8 days	(Ghosh et al. 2024)
5-aminolevulinic acid	Cold	Pepper, maize, tomato	Pot irrigation, leaf spray, seed soaking	Regulates stomatal opening; ROS and sucrose accumulation	25 ppm, 25 mg/L	3-days, 1 spray, seed soaking, 12 h	(Korkmaz et al. 2010; Wang et al. 2018b; Zhang et al. 2022c)
	Drought	Banana, cucumber	Leaf spray	Alleviates ROS	3 µM, 30 mg/L	2–3 sprays	(Li et al. 2011; Helaly et al. 2022)
	Heat	Cucumber	Leaf spray	Enhances antioxidant enzyme activities and soluble sugar accumulation	3 µM	2 sprays	(Zhang et al. 2012)
	Salt	Cucumber	Leaf spray	Increases expression of genes such as HEMA1 and CHLH	25 mg/L	2 sprays	(Wu et al. 2018)
Amino acids							
Glutamic acid	Salt	Tomato	Pot irrigation	Enhances mutualistic *Streptomyces globisporus* population	50 µg per plant (3 treatments)	3 times	(Kim and Kwak 2023)
Leucine	Heat	Chinese ginseng	Leaf spray	Modulates metabolites	3–5 mM	8 times	(Liu et al. 2023)
Aspartic acid	Salt	Wheat	Leaf spray	Alleviates ROS	0.4–0.8 mM		(Sadak et al. 2022)
Asparagine	Salt	Maize, wheat	Leaf spray	Enhances the activity of ROS scavengers	5–10 mM	4 times	(Wang et al. 2005; Kaya et al. 2013)
Proline	Salt	Moringa	Leaf spray	Improves antioxidant activity and decreases uptake of Na^+^ and Cl^−^	50 ppm	Every 15 days until pods dehisced	(El Moukhtari et al. 2020; Atteya et al. 2022)
Phenylalanine	Salt	Moringa	Leaf spray	Enhances osmoprotectants and stimulates antioxidant machinery	50 ppm	Every 15 days until pods dehisced	(Atteya et al. 2022)
	Salt	Arabidopsis	Pot irrigation	Regulates ABA signaling	0.3 mM		(Jakab et al. 2005)
	Drought	Arabidopsis, potato	Pot irrigation	Regulates stomatal closure and ABA signaling	0.3 mM	1 day	(Jakab et al. 2005; Sós-Hegedus et al. 2014)
Beta-aminobutyric acid (BABA)	Heat	Chinese cabbage	Leaf spray	Mitigates ROS; protects membrane	0.2 mM	5 sprays	(Quan et al. 2022
	Cold	Tobacco	Leaf spray	Decreases ROS production; increases antioxidant enzyme activities	0.1–1 mM	3 sprays	(Ma et al. 2020)
GABA	Drought	Creeping bentgrass	Leaf spray	Enhances accumulation of water-soluble carbohydrates and proline	0.5 mM	2 sprays	(Li et al. 2016)
	Heat	Creeping bentgrass, mungbean, rice	Pot irrigation, leaf spray	Improves turgor; upregulates osmoprotectants and antioxidants	1 mM	10 days, 2 sprays	(Nayyar et al. 2014; Li et al. 2016; Priya et al. 2019)
	Salt	Maize*,* mungbean, woodland tobacco, tomato	Pot irrigation, leaf spray	Strengthens antioxidant metabolism; increases amino acid metabolism; increases the expression of K^+^ transporter; regulates nitrogen metabolism and antioxidant potential	1–5 mM	5 sprays, 2 irrigations	(Akçay et al. 2012; Wu et al. 2020; Aljuaid and Ashour 2022; Ullah et al. 2023)
Poly γ-glutamic acid	Drought	Maize	Pot irrigation	Improves photosynthesis and rhizosphere microbial community	50 mg/L	5 days	(Ma et al. 2022)
	Salt	Rapeseed	Pot irrigation	Increases proline content and ROS scavengers	20 mg/L	2–6 days	(Lei et al. 2016)
Glycine betaine	Cold	Tomato	Leaf spray	Promotes desaturation process of lipids; increases membrane stability	10 mM	24 h	(Karabudak et al. 2014)
	Drought	Maiz, wheat	Leaf spray	Enhances ROS scavengers	3.65–3.84 g/L	1 spray	(Quan et al. 2004; He et al. 2011; Shafiq et al. 2021)
	Salt	Cucumber, okra	Leaf spray	Modulates salt uptake	50–100 mM	1–3 sprays	(Habib et al. 2012; Estaji et al. 2019)
Green tea catechins	Salt	Sweet pepper	Pot irrigation	Manages ROS and increase photosynthesis	2 mM (5 mL per plant)	1 time	(Yiu et al. 2012)
Glucosinolates	Drought	Arabidopsis	Leaf spray	Regulate stomatal closure	50 µM	6 sprays	(Salehin et al. 2019)
Humic acid	Heat	Arabidopsis	Liquid culture assay	Induces of HSPs family	860 mg/L	9 h	(Cha et al. 2020)
Nicotinic acid	Drought	Arabidopsis	Pot irrigation	Modulates metabolites, enhances energy level	5–10 mM	3-days	(Ahmad et al. 2021)
Putrescine	Cold	Arabidopsis, tomato	Leaf spray	Modulates ABA biosynthesis; regulates JA signaling	1 mM	1 spray	(Cuevas et al. 2008; Ding et al. 2021)
	Drought/osmotic	Wheat	Leaf spray	Increase amino acids and soluble sugars	0.1 mM	7 days	(Gupta et al. 2020; Doneva et al. 2021)
	Flooding	Welsh onion	Pot Irrigation	Mitigates oxidative stress	2 mM	24 h	(Yiu et al. 2009)
	Heat	Wheat	Leaf spray	Improves the total amino acid content	2.5 mM	2 sprays	(Hassanein et al. 2013)
	Salt	Belladonna	Seed soaking	Reduces salt accumulation	10 µM	8 h	(Ali 2000)
VOCs							
(*E*)-2-hexenal	Heat	Arabidopsis, tomato	Wet tissue	Induces heat stress related transcription factors	10 µM	1 time	(Yamauchi et al. 2015; Terada et al. 2017)
(*E*)-2-butenal	Heat	Arabidopsis	Wet tissue	Induces heat stress related transcription factors	10 µM	1 time	(Yamauchi et al. 2015)
(*E*)-3-hepten-2-one	Heat	Arabidopsis	Wet tissue	Induces heat stress related transcription factors	10 µM	1 time	(Yamauchi et al. 2015)
Eugenol	Heat	Tomato	Leaf spray	Modulates hormones like SA, JA	200 µg/mL	1 spray	(Tsai et al. 2019)
Z-3-HAC	Salt	Peanut	Leaf spray	Reduces ROS; improves photosynthesis and osmoregulation	200 µM	2 sprays	(Tian et al. 2019)
	Cold	Maize	Leaf spray	Up-regulates cold stress related genes	1 µg/mM	1 spray	(Cofer et al. 2018)

Non-proteinogenic amino acids, such as gamma-aminobutyric acid (GABA), beta-aminobutyric acid (BABA), glycine betaine, and 5-aminolevulinic acid, also play significant roles in abiotic stress responses (Sós-Hegedus et al. 2014; Cohen et al. 2016; Zulfiqar et al. 2019; Kaspal et al. 2021; Rhaman et al. 2021). Chemical treatments with BABA and GABA have been shown to enhance tolerance to drought, heat, cold, and salt stress (Jakab et al. 2005; Nayyar et al. 2014; Sós-Hegedus et al. 2014; Priya et al. 2019; Ma et al. 2020; Ullah et al. 2023; Yuan et al. 2023).

N-acetylglutamic acid (NAG), a non-proteinogenic amino acid and an intermediate in arginine metabolism, is synthesized from glutamic acid and acetyl-CoA by N-acetylglutamate synthase. Overexpression of *Solanum lycopersicum* N-acetylglutamate synthase 1 (*SlNAGS1*) in Arabidopsis alleviates drought and salt stress by accumulating intermediates of arginine metabolism. Moreover, exogenous NAG treatment enhances oxidative stress tolerance in Arabidopsis and rice by increasing histone acetylation of the *ZAT10* and *ZAT12* transcription factors for ascorbate peroxidases, resulting in reduced ROS accumulation (Hirakawa et al. 2023). NAG may contribute similarly to acetic acid in providing acetyl groups for histone modification.

Other metabolites, such as vanillic acid, improve salt stress tolerance, while exogenous glutathione application mitigates lead-induced oxidative stress in wheat. Maleic acid enhances metal chelation and antioxidant metabolism in *Brassica juncea* (Mahmud et al. 2017). Several additional metabolites also play significant roles in enhancing plant resilience to abiotic stress (Table 1).

## Glucosinolates

Glucosinolates (GLSs) are secondary metabolites predominantly found in the Brassicaceae family, playing a crucial role in protecting plants against pathogen attacks and herbivory, as well as regulating their response to drought stress (Salehin et al. 2019). GLSs comprise a core structure that includes a sulfated isothiocyanate (ITC) group linked to thioglucose and an R-group derived from amino acids (Halkier and Gershenzon 2006; Zhang et al. 2020). Based on the amino acid precursors, GLSs are classified into three major categories: aromatic, aliphatic, and indole GLSs (Halkier and Gershenzon 2006; Sønderby et al. 2010).

In Arabidopsis, approximately 40 distinct GLSs have been identified, highlighting their structural diversity (Halkier and Gershenzon 2006; Sønderby et al. 2010; Zhang et al. 2020). Disruptions in GLS biosynthesis can impair stomatal regulation. Interestingly, externally applied GLSs have been shown to restore normal stomatal function and enhance drought tolerance in mutants with auxin-sensitive Aux/IAA repressors deficient in GLS synthesis (Salehin et al. 2019). These findings underscore the potential of GLS treatment for improving drought stress tolerance (Salehin et al. 2019).

## Polyamines

Polyamines, including putrescine, spermidine, and spermine, are organic polycations characterized by the presence of more than two amino groups and variable hydrocarbon chains (Takahashi and Kakehi 2010). Pretreatment with putrescine has been shown to help osmotic stress-sensitive wheat varieties better tolerate adverse conditions (Doneva et al. 2021). At physiological pH, polyamines are positively charged and exhibit high electrostatic affinity for negatively charged molecules such as nucleic acids and proteins. These interactions enhance the stability of nucleic acids and enzyme activity (Takahashi and Kakehi 2010).

Polyamines are involved in a wide range of physiological processes in plants, from development to stress responses. Exogenous application of polyamines has been demonstrated to significantly enhance plant tolerance to various abiotic stresses, including drought, salinity, and extreme temperatures (Shao et al. 2022). Notably, thermospermine, an isomer of spermine, has been reported to improve salinity and heat stress tolerance in Arabidopsis (Sagor et al. 2013; Shinohara et al. 2019b). Thermospermine plays a role in repressing xylem differentiation (Takano et al. 2012), which may regulate sodium ion accumulation and contribute to improved salinity tolerance (Shinohara et al. 2019b). For a more comprehensive understanding of the role of polyamines in abiotic stress tolerance, readers are referred to Alcázar et al. (2020), González-Hernández et al. (2022), and Shao et al. (2022).

## Volatile organic compounds

Plants emit volatile organic compounds (VOCs) to interact with other plants, herbivores, pollinators, and microorganisms (Bouwmeester et al. 2019; Loreto and D’Auria 2022). The emission levels and compositions of VOCs, which vary with stress severity, provide insights into the activation of secondary metabolic pathways under stress. These compounds mediate complex interactions, modulating plant responses to a range of stresses (Baldwin et al. 2002; Bouwmeester et al. 2019; Loreto and D’Auria 2022). The production and emission of VOCs are tightly regulated by different stress conditions, influencing plant phenotype, metabolism, and defense mechanisms (Loreto and D’Auria 2022).

Bacteria also produce volatile compounds at low concentrations, which have broad-ranging effects. Both bacterial and plant-derived VOCs play critical roles in enhancing plant defense and improving agricultural productivity (Cellini et al. 2021). The processes of VOC emission and perception by neighboring plants are integral to plant communication. In plants, damaged cells and trichomes directly release VOCs, while in intact tissues, these compounds must traverse subcellular and plasma membranes, cell walls, cuticles, or air spaces to be emitted through stomata (Widhalm et al. 2023). VOC transport may occur via active or passive mechanisms. For passive diffusion, plants accumulate high levels of VOCs in cellular membranes, a process that can be energy-inefficient and potentially toxic (Widhalm et al. 2023).

Recent studies demonstrate that VOC perception by neighboring plants can stimulate airborne defenses. For example, methyl salicylate (MeSA) emitted by one plant is converted into salicylic acid by neighboring plants through the action of salicylic acid-binding protein-2 (SABP2), triggering a signaling cascade that activates the NAC2–SAMT1 module for MeSA biosynthesis, thereby inducing plant immunity (Gong et al. 2023). Green leaf volatiles (GLVs) are particularly important in priming plant defenses against herbivores and insects, functioning to either repel or attract them and their natural enemies (Bouwmeester et al. 2019).

Although VOC research has primarily focused on defense against biotic stress, evidence suggests that these compounds also regulate plant responses to various abiotic stresses (Cofer et al. 2018; Matsui and Engelberth 2022). For instance, (E)-2-hexenal, an eco-friendly GLV, exhibits strong antifungal activity and enhances heat stress tolerance in Arabidopsis by regulating heat stress-related transcription factors AtHSFA2 and AtMBF1c (Yamauchi et al. 2015). Similarly, treating peanut seedlings with Z-3-HAC activates antioxidant systems and promotes osmolyte accumulation, enhancing salinity stress tolerance (Tian et al. 2019). In tomato plants, spraying with eugenol, a VOC synthesized from phenylalanine, induces the expression of heat shock factors (HSFs) and heat shock proteins (HSPs), as well as increases salicylic acid levels, collectively improving thermotolerance (Tsai et al. 2019). Additionally, (E)-2-hexenal application mitigates heat shock injury in tomato seedlings (Terada et al. 2017). Other GLVs, such as (Z)-3-hexenyl-1-yl acetate (Z-3-HAC), have been shown to alleviate cold stress in maize seedlings and salinity stress in peanut plants (Tian et al. 2019).

Plant inoculation with plant growth-promoting bacteria (PGPB) enhances plant stress resistance and mitigates hypersensitive responses (Cellini et al. 2021). The use of plant growth regulators (PGRs) and microbe-based biotechnologies further improves drought stress tolerance in plants (Cellini et al. 2021). Enhanced resilience and yield preservation under drought stress are achieved through (1) enhancing osmotic adjustment capacity, (2) regulating antioxidant activity, and (3) protecting photosynthetic machinery to maintain efficiency (Zhang et al. 2022a). This is reflected in reduced rates of stress-induced volatile emissions, enabling stress assessment through non-invasive VOC measurement and improving resilience via VOCs emitted by PGPB (Sharifi and Ryu 2021; Chatterjee and Niinemets 2022). Elucidating the role of VOCs in abiotic stress responses offers significant potential for advancing research toward the development of stress-resilient crop plants.

## Nitric oxide, a gaseous molecule regulates signaling and stress tolerance

Nitric oxide (NO) is a gaseous signaling molecule that plays a multifaceted role in regulating diverse biological pathways, enabling plants to adapt to environmental stresses such as oxidative stress (Hartman et al. 2019; Mishra et al. 2021). NO interacts with reactive oxygen species (ROS), abscisic acid (ABA), ethylene, and jasmonic acid (JA) to modulate stress responses. For instance, the treatment of wheat leaves with the NO donor sodium nitroprusside (SNP) has been shown to reduce transpiration rates by inducing stomatal closure (Mata and Lamattina 2001). However, the direct regulation of stomatal closure by NO remains partially understood.

NO treatment inhibits photosynthesis by increasing intercellular CO_₂_ levels, which subsequently leads to stomatal closure (Mata and Lamattina 2001; Van Meeteren et al. 2020). This effect, however, is not observed under low CO₂ concentrations, suggesting that NO is not the sole factor contributing to NO-induced stomatal closure. Furthermore, in intact leaves, the rate of stomatal closure in response to NO is significantly slower than that induced by ABA application (Van Meeteren et al. 2020).

The exogenous application of S-nitrosoglutathione (GSNO), a major NO donor, has been observed to enhance stress tolerance in plants (Hu et al. 2017; Hasanuzzaman et al. 2020). Additionally, NO plays a critical role in regulating iron (Fe) homeostasis, particularly under Fe-deficient conditions, where it modulates key processes to maintain Fe balance (Tewari et al. 2021).

## Phytohormones and phytohormone agonists/antagonists regulate stress response and plant development

Phytohormones, including abscisic acid (ABA), brassinosteroids (BRs), jasmonic acid (JA), salicylic acid, and strigolactone, play crucial roles in regulating plant growth, development, and responses to various biotic and abiotic stresses (Table 2). While the exogenous application of these hormones has been shown to enhance stress tolerance in plants (Kinoshita et al. 2018; Vaidya et al. 2019), their use is often physiologically costly and may negatively impact plant growth and development (Kinoshita et al. 2018; Nemoto et al. 2018; Vaidya et al. 2019; Hayashi et al. 2023; Saito et al. 2023).

**Table 2:** Plant hormones improving abiotic stress resilience in plants

Compound	Stress	Plant species	Method	Mechanism	Concentration	Time	References
ABA	Drought	Arabidopsis	Leaf spray	Controls stomatal aperture	10–100 µM	1 spray	(Vaidya et al. 2019; Kinoshita et al. 2021; Zhang et al. 2021)
BR/EBL	Heat	Arabidopsis, rapeseed, tomato	MS media with 0.8–1% Agar	Enhances accumulation of heat shock proteins	1–10 µM	1 time	(Dhaubhadel et al. 1999; Kagale et al. 2007; Chen et al. 2022)
	Drought	Timor white gum	Leaf spray	Increases photosynthetic pigments; induces antioxidant system	50–100 nM	1 spray	(Barros et al. 2021)
Ethylene	Hypoxia/flooding	Arabidopsis	Injection with syringe	Reduces NO and ERFVII accumulation	~ 5 μl L^−1^	1 time	(Hartman et al. 2019, 2021)
MeJA	Salt	Desert cherry	Liquid culture assay	Increases antioxidase activity; maintains low sodium in roots and increases ABA biosynthesis	10–100 µM	5 times (every three days)	(Gao et al. 2021)
SA	Cold/freezing	Common bean, tomato, wheat	Pot irrigation, leaf spray, seed soaking	Increases the level of reduced glutathione and acts as an antioxidant	0.01–1 mM	20 ml pot irrigation/3 sprays / 24 h seed soaking	(Senaratna et al. 2000; Wang et al. 2018a)
	Drought	Common bean, tomato	Pot irrigation, leaf spray, seed soaking	Increases the level of reduced glutathione and acts as an antioxidant	0.1–0.5 mM	20 ml pot irrigation, 1 spray, 24 h seed soaking	(Senaratna et al. 2000)
	Heat	Common bean, tomato	Pot irrigation, leaf spray, seed soaking	Increases the level of reduced glutathione and acts as an antioxidant	0.1–0.5 mM	20 ml pot irrigation/1 spray / 24 h seed soaking	(Senaratna et al. 2000)
Strigolactone	Drought	Arabidopsis	Leaf spray	Regulates stomatal function and hormonal response pathways	5 µM	7 sprays	(Ha et al. 2014)
	Salt	Arabidopsis	Leaf spray		5 µM		(Ha et al. 2014)

To address these limitations, the development of hormone agonists and antagonists with improved binding efficiency and specificity has emerged as a promising approach. These compounds aim to enhance plant stress tolerance (Table 3) while minimizing adverse effects on growth and development, making them a focus of growing interest in plant stress biology research (Hagihara et al. 2019).

**Table 3:** Synthetic compounds improving abiotic stress resilience in plants

Compound	Stress	Plant species	Method	Mechanism	Concentration	Time	References
2,6-dihalopurines	Drought	Benghal dayflower	Liquid culture assay	Inhibits stomatal opening	50–100 µM	3 h	(Ueda et al. 2023)
3′-butyl ABA	Drought	Arabidopsis	Leaf spray	Inhibits PP2C and promotes stomatal closure	25 µM	2 sprays	(Yoshida et al. 2019, 2021)
3-cyclopropyl ABA	Drought	Arabidopsis	Leaf spray	Inhibits PP2C and promotes stomatal closure	25 µM	2 sprays	(Yoshida et al. 2019, 2021)
AMFs	Drought	Arabidopsis, soybean	Leaf pray	Promotes stomatal closure	10–50 µM	2 sprays	(Cao et al. 2017)
BITC, m-bis-BITC	Drought	Chrysanthemum, arabidopsis	Liquid culture assay	Inhibits stomatal opening	5–50 µM	3–24 h	(Aihara et al. 2023; Ueda et al. 2023)
FSL0260	Salt	Arabidopsis, rice	Liquid culture assay	Reduces ROS accumulation	20 µM	24 h	(Sako et al. 2020)
Indolyl-ethyl amine	Drought	Rice	Leaf spray	Inhibits ABA biosynthesis	20 µM	1 spray	(Vanitha et al. 2022)
iSB09	Drought	Arabidopsis	Leaf spray	Activates ABA receptor	50 µM	2 sprays	(Lozano-Juste et al. 2023)
Natolen128	Salt	Arabidopsis	Liquid culture assay	Regulates NO accumulation	2 µM	24 h	(Sako et al. 2021c)
Opabactin	Drought	Arabidopsis, tomato, wheat	Leaf spray	Mimics ABA singaling	50–100 µM	1 spray	(Vaidya et al. 2019; Vaidya et al. 2021)
SCLs	Drought	Benghal dayflower	Leaf spray	Suppresses stomatal opening	20–50 µM	1 spray	(Toh et al. 2018)
KM	Drought	Barrelclover	Leaf spray	Regulates ROS and RNS	100 µM	1 spray	(Filippou et al. 2016)
	Salt	Barrelclover	Leaf spray	Regulates of sugars and amino acids accumulation	100 µM	1 spray	(Filippou et al. 2016)

## ABA and chemicals regulate stomatal opening and drought stress tolerance

Stomata, tiny pores located on the aerial parts of plants, are essential for regulating transpiration and optimizing CO₂ uptake for photosynthesis (Hewage et al. 2020). Abscisic acid (ABA) plays a critical role in controlling stomatal opening, influencing water potential, root water uptake, and transpiration (Juenger and Verslues 2023). While stomatal closure aids in water conservation and enhances drought stress tolerance, it also limits CO₂ absorption and increases photorespiration, creating a trade-off with photosynthetic efficiency (Yang et al. 2019).

Chemical treatments with ABA can effectively induce stomatal closure; however, their application can be costly and may hinder plant growth (Hewage et al. 2020; Kinoshita et al. 2021). Developing specific agonists and antagonists to regulate stomatal activity has emerged as a promising strategy. In Arabidopsis, 14 PYR/PYL/RCAR ABA receptors bind to ABA, inhibiting PP2C-mediated dephosphorylation of downstream protein kinases, thereby regulating stomatal opening and other physiological responses (Yang et al. 2019; Shinozaki and Yamaguchi-Shinozaki 2022). Recent advancements in chemical treatments with ABA agonists and antagonists have demonstrated significant improvements in plant water-use efficiency (Vaidya et al. 2019; Yoshida et al. 2021; Lozano-Juste et al. 2023). Beyond drought stress tolerance, ABA also regulates seed germination inhibition, growth control, senescence, and immune responses (Raghavendra et al. 2010; Hewage et al. 2020).

The development of ABA agonists that specifically target stomatal regulation without adversely affecting seed germination or plant growth is crucial for enhancing crop production. Chemical genetic approaches have identified synthetic molecules that modulate stomatal movement. For example, screening a chemical library created via C–H amination reactions revealed a stomata-influencing molecule (SIM) (Toda et al. 2022). SIM1 inhibits light-induced stomatal opening in dayflower. This discovery provides opportunities to modify SIM molecules to regulate stomatal dynamics and enhance drought stress tolerance (Toda et al. 2022).

Stomatal opening is stimulated by light through the phosphorylation of plasma membrane (PM) H⁺-ATPases, activated by intracellular signal transduction pathways in guard cells. This mechanism generates the primary force for stomatal opening (Aihara et al. 2023; Ueda et al. 2023). Small molecules such as 2,6-dihalopurines and benzyl isothiocyanate (BITC) inhibit PM H⁺-ATPase phosphorylation, thereby reducing stomatal opening. BITC derivatives with multiple isothiocyanate groups (multi-ITCs) are more effective in minimizing stomatal opening over extended periods (Aihara et al. 2023). ABA ANTAGONIST1 (AA1), which targets all ABA receptors and blocks ABA signaling, has been observed to delay leaf senescence in both Arabidopsis and rice (Ye et al. 2017).

Stomatal development influences not only water-use efficiency but also overall crop productivity (Shinozaki and Yamaguchi-Shinozaki 2022). Synthetic chemicals identified through forward genetic screens have shown potential for increasing stomatal density, thereby enhancing plant productivity in Arabidopsis (Kinoshita et al. 2021).

## Forward and reverse genetic screening of ABA regulators

Forward genetic screens identify chemicals that enhance tolerance to specific stresses, while reverse genetic approaches focus on chemicals that interact with specific receptors or proteins (Ito et al. 2015). With the structural and functional characteristics of ABA receptors well understood, virtual screening has become an effective tool for discovering chemicals that bind to these receptors. These efforts aim to identify chemicals capable of temporally and spatially regulating specific ABA receptors, inducing drought stress tolerance without impairing plant growth (Dejonghe et al. 2018; Yoshida et al. 2019, 2021; Vanitha et al. 2022).

Screening for ABA agonists that specifically target one or more of the 11 ABA receptors revealed that (+)-3′-alkyl ABAs act as receptor-specific agonists (Yoshida et al. 2019). Among these, (+)-3′-butyl ABA induces strong transcriptional responses and stomatal closure with minimal effects on seed germination and plant growth (Yoshida et al. 2019). The addition of a cyclopropyl group at position 3 of ABA enhances binding specificity by occupying the C6 cleft in the receptor’s ABA-binding pocket. Plants treated with 3′-butyl ABA exhibit superior drought tolerance compared to those treated with 3-cyclopropyl derivatives (Yoshida et al. 2021).

## Advances in chemical and protein engineering

Enhancing the interaction between agonists and conserved lysine residues in ABA receptors has shown promise for regulating stomatal activity over extended periods. Virtual screening identified opabactin as a potent regulator of stomatal opening. Treatment with opabactin in monocot and eudicot plants has demonstrated enhanced drought stress tolerance, marking a significant advancement in developing next-generation agrochemicals (Vaidya et al. 2019).

Other compounds, such as indolyl-ethyl amine and serotonin, inhibit ABA biosynthesis by temporarily regulating the bZIP23 transcription factor (Vanitha et al. 2022). These chemicals boost seed germination in rice, wheat, and soybean, even in the presence of ABA, and enhance photosynthesis while conserving water by reducing the transcription levels of bZIP23 and its target genes, such as *NCED4*, *PP2C49*, and *CO*_*3*_. Under mild drought stress, these molecules improve stomatal conductance, spikelet fertility, and yield in rice (Vanitha et al. 2022). In addition, several other identified chemicals have been shown to regulate stomatal opening and drought stress tolerance without negatively impacting plant growth (Cao et al. 2017; Nemoto et al. 2018).

Combining chemical and protein engineering strategies could further improve plant stress tolerance. For instance, modifying ABA receptors to enhance ligand recognition has produced promising results. The engineered *CsPYL1* ABA receptor (*CsPYL15m*) binds more efficiently to the ABA agonist iSB09, activating ABA signaling pathways and enhancing drought tolerance in Arabidopsis without compromising growth (Lozano-Juste et al. 2023). Similarly, introducing fluorine atoms into the benzyl ring of the ABA agonist AM1 improves its binding affinity to ABA receptors (Cao et al. 2017). These modified chemicals, known as AMFs, form additional hydrogen bonds with residues in the receptor’s ligand-binding pocket (Cao et al. 2017). AMFs effectively close stomata for extended periods and induce the expression of stress-responsive genes. Application of AMFs to transgenic plants overexpressing the ABA receptor *PYL2* has conferred improved drought tolerance in both Arabidopsis and soybean (Cao et al. 2017). The development of these innovative chemicals represents a significant step toward commercializing agrochemicals that regulate stomatal closure and enhance stress tolerance in crops.

## Brassinosteroids regulate plant growth, development, and stress response

Brassinosteroids (BRs) are pivotal in promoting plant growth and development (Kim and Russinova 2020). Chemical treatments with BRs have demonstrated efficacy in enhancing stress tolerance, particularly salinity stress, by minimizing Na⁺ uptake and regulating ROS production. Plants treated with BRs show increased tolerance to cold, heat, drought, and salt stress (Jin et al. 2015; Fu et al. 2019; Chen et al. 2022). Notably, BRs enhance salinity and heat stress tolerance through ethylene and salicylic acid signaling pathways, respectively (Divi et al. 2010; Tao et al. 2015; Zhu et al. 2016). The interaction of BRs with other hormone signaling pathways plays a critical role in augmenting stress tolerance (Planas-Riverola et al. 2019).

For instance, the exogenous application of 24-epibrassinolide (EBL) improves heat stress tolerance in Arabidopsis, rapeseed, and tomato seedlings (Dhaubhadel et al. 1999; Kagale et al. 2007; Chen et al. 2022). Treatment with 100 nM EBL in *Eucalyptus urophylla* mitigates water deficiency by enhancing antioxidant enzyme activities, electron flux, chloroplast pigments, PSII efficiency, and overall photosynthesis (Barros et al. 2021). Additionally, BRs regulate Fe uptake and translocation in rice, with BR-treated plants exhibiting increased tolerance to Fe toxicity (Wang et al. 2015; Tadaiesky et al. 2021). These findings highlight the potential of BRs to mitigate abiotic stresses and improve crop productivity.

## Ethylene

Ethylene, a gaseous hormone, plays a vital role in plant adaptation to stress conditions, particularly hypoxia stress, and interacts with molecules such as nitric oxide (NO). Plants detect submergence by trapping ethylene, which facilitates their adaptation to hypoxic conditions (Hartman et al. 2019). Ethylene interacts with the NO scavenger Phytoglobin 1, stabilizing ethylene response factor VII (ERFVII) before hypoxia occurs. This ethylene-mediated depletion of NO and subsequent ERFVII accumulation primes plants to survive subsequent hypoxic stress (Hartman et al. 2019).

The interplay of NO, ROS, and ethylene is crucial for regulating flooding responses, enabling plants to mitigate stress under submerged conditions (Hartman et al. 2019). Additionally, natural variations in ethylene production contribute to stress response regulation, priming plants for survival under hypoxia (Fukao et al. 2006; van Veen et al. 2013).

## Jasmonic acid (JA): regulation of plant immunity and stress response

Jasmonic acid (JA) serves dual functions in regulating plant immunity and drought stress responses (Howe et al. 2018). Plants synthesize JA-Ile in response to herbivory and pathogen infections, triggering defense responses that often come at the cost of plant growth and development (Vincent et al. 2022; Hayashi et al. 2023; Saito et al. 2023). The COI1-JAZ co-receptor complex perceives JA-Ile, leading to the degradation of JAZ proteins. This degradation releases transcription factors such as MYC2/3/4, initiating genome-wide transcriptional changes that drive jasmonate responses (Howe et al. 2018).

Exogenous application of methyl jasmonate (MeJA) enhances defense responses and mitigates stress damage by modulating oxidative stress, although it often limits plant growth (Wasternack and Hause 2013). MeJA treatment increases the accumulation of osmolytes and regulates Na⁺/K⁺ ratios (Gao et al. 2021). Additionally, MeJA triggers the synthesis and signaling of ABA and JA, leading to transcriptomic changes that enhance stress responses (Gao et al. 2021). However, while MeJA treatment prioritizes defensive responses, it exacerbates growth inhibition under salt stress (Gao et al. 2021).

Interestingly, acetic acid treatment activates JA signaling pathways, but chemical treatments with JA have not been shown to enhance drought stress tolerance (Kim et al. 2017; Kudo et al. 2023). Compared to its well-documented role in biotic stress responses, JA's function in abiotic stress tolerance remains less explored. Further research is needed to elucidate the mechanisms underlying JA's role in drought and other abiotic stress conditions.

## Salicylic acid regulates plant growth, development, and stress response

Salicylic acid (SA) plays a crucial role in regulating plant growth, development, ion transport, photosynthesis, and water transpiration, often interacting with other plant hormones (Jiang et al. 2013; Koo et al. 2020). As a defense-related hormone, SA enhances resistance against various microbial pathogens, including viruses, bacteria, fungi, and oomycetes (Kunkel and Brooks 2002; Koo et al. 2020). The exogenous application of SA modulates genes responsive to abiotic stress, activating the antioxidant system and enhancing tolerance to stresses such as cold, heat, salinity, heavy metals, and nutrient deficiencies (Koo et al. 2020; Sako et al. 2021b).

In addition to activating the antioxidant system, SA interacts with other hormonal pathways, regulates mineral uptake, facilitates osmolyte accumulation, scavenges reactive oxygen species, and contributes to the synthesis of secondary metabolites, all of which play significant roles in improving abiotic stress tolerance (Koo et al. 2020).

## Strigolactone regulates plant growth and development

Strigolactone is a key hormone that optimizes plant growth and development, particularly under stress conditions, enabling plants to compete effectively with neighboring organisms for limited resources (Li et al. 2020). Phosphate deficiency triggers an increase in strigolactone levels, which modifies root architecture and promotes fungal symbiosis, enhancing phosphate absorption (Umehara et al. 2010; Balzergue et al. 2011). Strigolactones have also been implicated in responses to abiotic stresses such as drought (Brewer et al. 2013; Lumba et al. 2017; Aliche et al. 2020; Li et al. 2020).

Karrikins (KARs), smoke-derived structural analogs of strigolactones, influence plant growth and drought responses (Li and Tran 2015; Li et al. 2017). While several other hormones contribute to plant growth and stress resilience (Santner et al. 2009), this review focuses on the potential of phytohormones, including strigolactones, in priming plants to enhance stress resilience and growth.

## Phytohormones and agricultural applications

Phytohormones interact with each other to regulate complex signaling and metabolic pathways. Spatial and temporal regulation of phytohormones is, therefore, essential for mitigating stress responses and improving crop production. The development of agonists and antagonists targeting specific receptors holds potential as cost-effective solutions for commercial applications, offering a means to balance trade-offs and enhance crop productivity.

## Synthetic compounds

High-throughput screening of chemical libraries has identified several synthetic compounds with potential for enhancing abiotic stress tolerance (Table 3). FSL0260, sourced from the NPDepo chemical library, improves salt stress tolerance in Arabidopsis and rice by inhibiting complex I of the mitochondrial electron transport system, which activates the mitochondrial alternative respiratory system. This process reduces reactive oxygen species (ROS) accumulation under high salt stress, thereby enhancing plant tolerance (Sako et al. 2020).

Natolen128, identified from the Institute of Transformative Bio-Molecules (ITbM) chemical library, has also been shown to enhance salt stress tolerance in Arabidopsis, likely by regulating nitric oxide (NO) accumulation (Sako et al. 2021b). Additionally, chemical screening has identified compounds that regulate stomatal movement. For example, stomatal closing compounds (SCLs) inhibit light-induced stomatal opening by disrupting signaling between the phototropin receptor and the PM H⁺-ATPase enzyme. SCL1 has been shown to enhance drought stress tolerance (Toh et al. 2018).

Recent studies have identified benzyl isothiocyanate (BITC) from the International Drug Collection (MicroSource Discovery System) as a potent inhibitor of stomatal opening, suppressing PM H⁺-ATPase phosphorylation. BITC derivatives, such as m-bis-BITC, have demonstrated prolonged inhibition of stomatal opening and reduced leaf wilting, further supporting their potential for enhancing drought stress tolerance (Aihara et al. 2023).

Kresoxim-methyl (KM), a fungicide, has been shown to regulate various physiological and developmental processes in plants. Pretreatment of *Medicago truncatula* with KM enhances tolerance to drought and salt stresses, as evidenced by improved physiological parameters. KM treatment promotes proline biosynthesis, modulates reactive oxygen and nitrogen species signaling, and minimizes cellular damage under stress conditions (Filippou et al. 2016). Ongoing screening of chemical libraries, through both forward and reverse genetic approaches, is expected to yield novel compounds capable of significantly enhancing agricultural productivity under changing environmental conditions.

## Peptide signaling

Plant development relies significantly on intercellular signaling mediated by peptide hormones and membrane-localized receptor kinases. These interactions play crucial roles in various cellular functions, modulating peptide signaling through receptor binding (Takahashi and Shinozaki 2019). Developing agonistic or antagonistic approaches offers promising opportunities for agricultural applications. Small molecules are known to compete with and displace natural ligands in binding to peptide hormones.

A systematic high-throughput screening method, employing bead-immobilized receptor kinases and fluorescent-labeled peptide ligands, has proven effective in identifying molecules that bind to peptide hormones (Shinohara et al. 2019a). This innovative technique enables the discovery of small molecules that competitively bind to peptide hormone receptors, displacing natural ligands. Screening approximately 30,000 chemicals against the Arabidopsis CLE9-BAM1 ligand-receptor pair identified NPD12704 as a molecule capable of binding to BAM1 and inhibiting CLV3's interaction with BAM1 (Shinohara et al. 2019a). Notably, NPD12704 exhibits minimal interference with the binding of CLV3 to CLV1, the closest homolog of BAM1, demonstrating its preferential specificity for BAM1. In *Clv1-101* mutant plants, treatment with NPD12704 resulted in an enlarged shoot apical meristem phenotype (Shinohara et al. 2019a).

These findings establish a valuable technological framework for identifying small non-peptide chemicals that precisely regulate receptor kinase-mediated peptide hormone signaling, providing a means to control plant growth. The small peptide CLE25, synthesized in the vascular tissues of Arabidopsis, moves from roots to leaves and interacts with BAM receptors to regulate the plant’s dehydration response (Takahashi et al. 2018). Exogenous application of CLE25 modulates dehydration responses (Takahashi et al. 2018, 2019). Similarly, AtPep3, a hormone-like peptide, has been shown to regulate salinity stress responses. External application of AtPep3 enhances salt stress tolerance in Arabidopsis (Nakaminami et al. 2018).

## Epigenetic regulators empower plants for stress tolerance

DNA methylation, regulatory RNAs (including noncoding RNAs, both modified and unmodified), chromatin remodeling, histone variants, and histone modifications collectively form a complex epigenetic regulatory network that operates in a coordinated manner (Goldberg et al. 2007; Kinoshita and Seki 2014; Kim et al. 2015; Matsui and Seki 2022). Enzymes responsible for DNA methylation and histone modifications have emerged as promising targets for drug discovery, driven by the rising demand for clinical therapies addressing cancer and cardiovascular diseases (Baylin 2005; Kelly et al. 2010). A range of epigenetic inhibitors targeting enzymes involved in DNA methylation, histone acetylation, and histone methylation are currently undergoing clinical trials (Lopez et al. 2022). Moreover, natural and synthetic compounds that alter epigenetic states have been shown to affect abiotic stress responses in plants (Table 4).

**Table 4:** Epigenetic compounds improving abiotic stress resilience in plants

Compound	Stress	Plant species	Method	Mechanism	Concentration	Time	References
Sodium butyrate	Salt	Arabidopsis	Liquid culture assay	Inhibits histone deacetylase	1 mM	16 h	(Ueda et al. 2017)
Trichostatin A	Salt	Arabidopsis	Liquid culture assay	Inhibits histone deacetylase	5 µM	16 h	(Ueda et al. 2017)
SAHA	Salt	Arabidopsis, cassava cotton	Liquid culture assay	Inhibits histone deacetylase	100 µM	16–24 h	(Patanun et al. 2017; Ueda et al. 2017; He et al. 2020)
Romidepsin (FK228)	Salt	Arabidopsis	Liquid culture assay	Inhibits histone deacetylase	5 µM	16 h	(Ueda et al. 2017)
MS-275	Salt	Arabidopsis	Liquid culture assay	Inhibits histone deacetylase	100 µM	16 h	(Ueda et al. 2017)
LBH-589	Salt	Arabidopsis	Liquid culture assay	Inhibits histone deacetylase	5 µM	16 h	(Ueda et al. 2017)
JNJ-26481585	Salt	Arabidopsis	Liquid culture assay	Inhibits histone deacetylase	5 µM	16 h	(Ueda et al. 2017)
MC1293	Salt	Arabidopsis	Liquid culture assay	Inhibits histone deacetylase	100 µM	16 h	(Ueda et al. 2017)
Ky-2	Salt	Arabidopsis	Liquid culture assay	Increases H4 acetylation at AtSOS1	1 µM	24 h	(Sako et al. 2016)
Ky-9, Ky-72	Salt	Arabidopsis	Liquid culture assay	Increasing H3 acetylation and upregulates genes related to salinity tolerance	1 µM	24 h	(Nguyen et al. 2018)
Zebularine	Heat	Arabidopsis (Histone H1 mutant background)	Liquid culture assay	Inhibits DNA methly transferase	40 µM	10 days	(Liu et al. 2021)

## Histone deacetylase (HDAC) inhibitors and abiotic stress tolerance

Among epigenetic modifiers, histone deacetylase (HDAC) inhibitors have demonstrated particular efficacy in enhancing salinity stress tolerance. HDACs fine-tune acetylation levels in conjunction with histone acetyltransferases (Shahbazian and Grunstein 2007). Treatments with various HDAC inhibitors, including Chlamydocin-hydroxamic acid analogs (Ky-2, -9, -72) (Nishino et al. 2004), FK228 (Nakajima et al. 1998), JNJ-26481585 (Arts et al. 2009), LBH589 (Scuto et al. 2008), MC1293 (Hamalainen et al. 2008), MS-275 (Saito et al. 1999), sodium butyrate (NaBT) (Boffa et al. 1978), and trichostatin A (TSA) (Yoshida et al. 1990), have improved salinity stress tolerance in Arabidopsis seedlings (Sako et al. 2016; Ueda et al. 2017; Ueda et al. 2018; Nguyen et al. 2018). Interestingly, Arabidopsis plants deficient in HDA19, a specific HDAC isoform, exhibit enhanced tolerance to multiple abiotic stresses, including salinity, drought, and heat (Ueda et al. 2017). Additionally, treatment of crops with SAHA, another HDAC inhibitor, has increased salinity stress tolerance in cassava (Patanun et al. 2017) and cotton (He et al. 2020).

NaBT is endogenously produced, while FK228 and TSA are fungal-derived natural compounds that target HDAC enzymatic activity. Their structures have been modified to enhance selectivity, reduce metabolic instability, lower retention, and minimize nonspecific toxicity in human cells. Synthetic compounds, including JNJ-26481585, LBH-589, MC1293, MS-275, and SAHA, have undergone improvements for better efficacy and safety. Alongside synthetic development, natural products such as apigenin (Pandey et al. 2012) and luteolin (Attoub et al. 2011), which inhibit human HDAC enzymatic activity, continue to be explored (Rajaselvi et al. 2023). Although the potential for these phytochemical compounds to enhance abiotic stress tolerance in plants remains uncertain, metabolic manipulation to accumulate exogenous compounds with HDAC inhibitory functions represents a promising strategy. For instance, apigenin accumulation has been shown to protect plants against UV-B-induced damage (Righini et al. 2019).

Most HDAC inhibitors bind to the active-site zinc ion within HDAC proteins, leading to enzymatic inactivation. For practical field applications aimed at enhancing stress resilience, plant-specific HDAC inhibitors are critical. Compounds that bind to non-active sites could increase species-specific selectivity, making them more suitable for agricultural use.

## DNA and histone methylation in abiotic stress responses

In contrast to HDAC inhibitors, there is limited evidence that plants treated with compounds altering DNA or histone methylation exhibit increased tolerance to abiotic stress. However, in mutant backgrounds, Arabidopsis linker histone H1-deficient plants (*h1.1–1/h1.2–2* double mutants) treated with the DNA methyltransferase inhibitor zebularine showed enhanced heat stress tolerance (Liu et al. 2021). These findings suggest that combining DNA methylation inhibitors with compounds capable of depleting histone H1 variants may increase heat stress tolerance in plants.

Previous studies have established that epigenetic elements, including histone methylation, play critical roles in abiotic stress responses. Components and enzymes involved in histone methylation, such as histone methyltransferases and demethylases, contribute to plant resilience under stress conditions (Nunez-Vazquez et al. 2022). This indicates potential for improving abiotic stress tolerance by modifying histone methylation levels.

The development of novel inhibitors targeting both histone methyltransferases and demethylases is ongoing (Zhao and Shilatifard 2019). These inhibitors could provide an avenue for modulating abiotic stress responses by altering histone methylation states, representing a promising strategy for enhancing plant resilience in changing environmental conditions.

## Conclusion and prospects

**Fig. 4:**
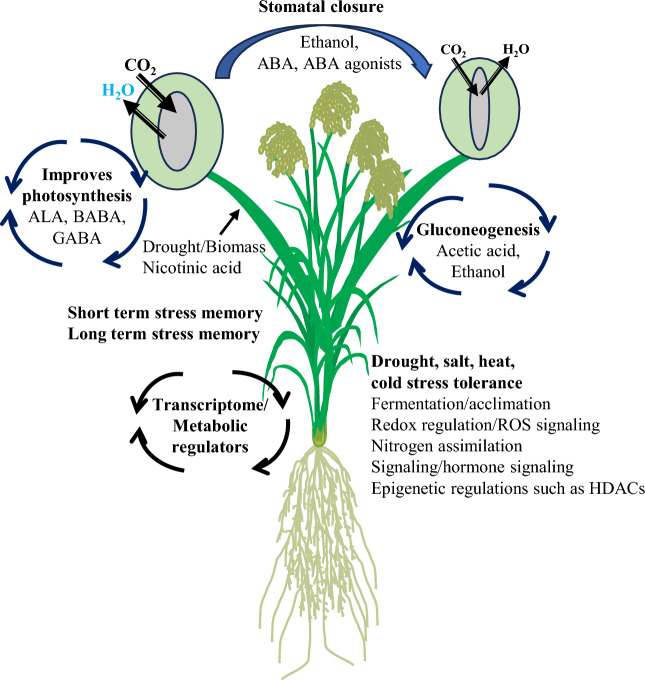
Chemical application improves stress resilience in plants. Chemical treatments using various metabolites, hormones, synthetic compounds, and epigenetic regulators induce metabolic, molecular, physiological, and morphological changes that enhance stress resilience in plants.

In recent years, chemical treatment has emerged as an effective tool for mitigating environmental stresses and enhancing crop production. Various agents, including phytohormones, agonists, antagonists, epigenetic regulators, and plant metabolites, have demonstrated significant potential in augmenting stress tolerance across diverse crop species (Fig. 4). Notably, several epigenetic inhibitors that modulate DNA methylation, histone acetylation, and histone methylation present promising opportunities for developing stress-resilient crops.

Effective chemical treatment strategies should emphasize cost-effectiveness, environmental sustainability, and ease of application. To achieve this, coordinated efforts are essential to optimize the dose, duration, and method of chemical application, whether foliar or via irrigation, tailored to specific plant species. These strategies must also consider the intensity, duration, and type of environmental stress. The concentration of chemicals and their mode of application may vary depending on the stress type, plant species, and growth stage.

A critical aspect of stress management involves the regulation of stomatal opening, which plants modulate differently under heat and drought stress. Under heat stress, plants open their stomata to enhance transpiration and lower leaf temperatures (Gommers 2020). In contrast, drought stress typically induces stomatal closure to conserve water, leading to elevated leaf temperatures (Gupta et al. 2012). This contrasting physiological response poses a unique challenge, as plants that mitigate drought stress by reducing transpiration may become more susceptible to heat stress.

In field conditions, heat and drought stresses often occur simultaneously, particularly in summer crops, necessitating the development of integrated strategies to address both stresses. Although ethanol priming has been shown to enhance tolerance to drought, heat, and salt stress, field trials combining these approaches are required to validate their efficacy in improving crop production under increasingly adverse environmental condsitions. Despite these challenges, chemical treatment offers novel avenues for deploying sustainable technologies to enhance crop productivity and ensure food security.

## Data Availability

Enquiries about data availability should be directed to the authors.
