# Correction: Chemical application improves stress resilience in plants

**DOI:** 10.1007/s11103-025-01621-6

**Published:** 2025-07-23

**Authors:** Khurram Bashir, Daisuke Todaka, Kaori Sako, Minoru Ueda, Farhan Aziz, Motoaki Seki

**Affiliations:** 1 Plant Genomic Network Research Team, RIKEN Center for Sustainable Resource Sciences, 1-7-22 Suehiro-cho, Tsurumi-ku, Yokohama, Kanagawa 230-0045 Japan; 2 Department of Life Sciences, SBA School of Science and Engineering, Lahore University of Management Sciences, DHA Phase 5, Lahore, Pakistan; 3 Department of Advanced Bioscience, Faculty of Agriculture, Kindai University, Nakamachi, Nara 3327-204 Japan; 4 Plant Epigenome Regulation Laboratory, RIKEN Cluster for Pioneering Research, 2-1 Hirosawa, Wako, Saitama Japan; 5 Kihara Institute for Biological Research, Yokohama City University, Yokohama, Japan; 6 Graduate School of Science and Engineering, Saitama University, Saitama, Saitama Japan

**Correction: Plant Molecular Biology (2025) 115:47**

10.1007/s11103-025-01566-w

In the above mentioned publication the funding section was scrambled and was incorrect. The original article has been corrected and the funding section is also published here.

